# A large-scale multi-environment study dissecting adult-plant resistance haplotypes for stripe rust resistance in Australian wheat breeding populations

**DOI:** 10.1007/s00122-025-04859-2

**Published:** 2025-03-13

**Authors:** Natalya Vo Van-Zivkovic, Eric Dinglasan, Jingyang Tong, Calum Watt, Jayfred Goody, Daniel Mullan, Lee Hickey, Hannah Robinson

**Affiliations:** 1 Centre for Crop Science, Queensland Alliance for Agriculture and Food Innovation, The University of Queensland, St Lucia, QLD Australia; 2 Centre for Animal Science, Queensland Alliance for Agriculture and Food Innovation, The University of Queensland, St Lucia, QLD Australia; 3 InterGrain Pty Ltd, Perth, WA 6163 Australia

## Abstract

**Key message:**

Genetic variation in stripe rust resistance exists in Australian wheat breeding populations and is environmentally influenced. Stacking multiple resistance haplotypes or using whole-genome approaches will improve resistance stability and environmental specificity.

**Abstract:**

Wheat stripe rust (*Puccinia striiformis*) is a fungal disease responsible for substantial yield losses globally. To maintain crop productivity in future climates, the identification of genetics offering durable resistance across diverse growing conditions is crucial. To stay one-step ahead of the pathogen, Australian wheat breeders are actively selecting for adult-plant resistance (APR), which is considered more durable than seedling resistance. However, deploying resistance that is stable or effective across environments and years is challenging as expression of underling APR loci often interacts with environmental conditions. To explore the underlying genetics and interactions with the environment for stripe rust resistance, we employ haplotype-based mapping using the local GEBV approach in elite wheat breeding populations. Our multi-environment trial analyses comprising 35,986 inbred lines evaluated across 10 environments revealed significant genotype-by-environment interactions for stripe rust. A total of 32 haploblocks associated with stripe rust resistance were identified, where 23 were unique to a specific environment and nine were associated with stable resistance across environments. Population structure analysis revealed commercial or advanced breeding lines carried desirable resistance haplotypes, highlighting the opportunity to continue to harness and optimise resistance haplotypes already present within elite backgrounds. Further, we demonstrate that *in silico* stacking of multiple resistance haplotypes through a whole-genome approach has the potential to substantially improve resistance levels. This represents the largest study to date exploring commercial wheat breeding populations for stripe rust resistance and highlights the breeding opportunities to improve stability of resistance across and within target environments.

**Supplementary Information:**

The online version contains supplementary material available at 10.1007/s00122-025-04859-2.

## Introduction

Wheat (*Triticum aestivum*) is an essential crop that feeds almost 40% of the global population (Waqar et al. 2018). Over 778 million tons are produced annually (Grote et al. 2021; Statista 2023), providing over USD$150 billion to the global economy (Expert Market Research 2022). Wheat production faces a multitude of stress factors that increasingly threaten yields worldwide (Figueroa et al. 2018). Monocropping of genetically homogenous populations, and the genetic uniformity that ensued, has expedited the prevalence and severity of pathogen attack (Shiferaw et al. 2013; Solh et al. 2012) which, in recent years, has further been compounded by climate change and globalisation (Shiferaw et al. 2013).

Wheat fungal diseases are obligate biotrophs that cause 15–20% of yield loss per year (Figueroa et al. 2018). Stripe rust (*Puccinia striiformis*) or yellow rust (YR) is the most economically important wheat fungal rust disease found in over 60 countries (Carmona et al. 2020; Chen 2020; Chen and Kang 2017; Schwessinger 2017; Wellings 2011). This pathogen has potential to cause up to 100% in yield losses in the 88% of susceptible cultivars that underpin global wheat population (Beddow et al. 2015; Waqar et al. 2018). Grain yield and quality are markedly reduced by YR infection as the biotrophic nature of this fungi diverts energy away from its host to promote its own life cycle, causing extensive necrotic damage and reducing photosynthetic capacity of the crop (Figueroa et al. 2018).

Breeding for genetic resistance is widely considered the most sustainable and effective way to mitigate YR epidemics and subsequent yield losses (Carmona et al. 2020; Chen 2020; Solh et al. 2012). Wheat YR resistance can be broadly classified into two types: all-stage resistance (ASR) and adult-plant resistance (APR). Traditionally, wheat breeders routinely selected for ASR genes, where a single gene can provide high levels of resistance through induction of a race-specific hypersensitivity response that blocks pathogen sporulation and disease development (Abhinandan et al. 2018; Chen et al. 2014; Chen 2020; Chen and Kang 2017; Ellis et al. 2014; Jamil et al. 2020; Schwessinger 2017; Wang et al. 2019). However, the resulting incompatible host/pathogen interaction creates a selection pressure which drives stepwise evolution in the pathogen resulting in resistance breakdown of ASR genes from the emergence of new virulent races (Chen 2020; Chen and Kang 2017; Kumar et al. 2020; Wang et al. 2023). Consequently, once widely cultivated wheat varieties containing *Yr2*, *Yr9*, *Yr10*, *Yr17*, *Yr24*/*Yr26* and *Yr27* ASR genes have since broken down, leading to devastating global YR epidemics (Chen et al. 2014; Chen 2020; McIntosh et al. 2018). The continuous appearance of new avirulence/virulence combinations has led to extensive pathogen variability and the existence of multiple geographically distinct pathotypes (Chen and Kang 2017; Wellings 2007) best seen in Australia where at least 20 new pathotypes have arisen from single gene mutations since its introduction to the continent (Chen and Kang 2017; Solh et al. 2012; Wellings 2007).

To combat this, Australian wheat breeders have actively selected for quantitative forms of resistance including APR, which is a non-race-specific resistance that becomes most effective at adult-plant growth stages. Although a single APR gene provides a non-hypersensitive, weak level of resistance that only acts to inhibit YR growth and spore production, when these “slow rusting” genes are pyramided within a single individual, a high level or “near-immune” response can be achieved through their additive effects (Bariana et al. 2007a; Chen et al. 2014; Chen and Kang 2017; Schwessinger 2017; Wang et al. 2023, 2019; Waqar et al. 2018). Historically, Australian breeders have predominately used a small set of *Yr* genes within breeding (e.g. *Yr9*, *Yr18*, *Yr27*, *Yr33* and *Yr34*) and now with a focus on enhanced durability against evolving pathogens often a combination of APR and ASR genes (e.g. *Yr18*, *Yr29*, *Yr46*) is favoured in current breeding efforts. The challenge lies in understanding the APR x E (environment) interaction, where resistance can be less effective under certain growing conditions (e.g. cooler conditions) or specific circumstances like early onset of the disease, while the crop is at late stages of tillering. Consequently, diverse haplotypes may be necessary to confer robust resistance in varying environments, posing a persistent challenge for breeders in developing varieties with stable resistance across diverse growing conditions.

To date, over 80 *Yr* genes have been catalogued and over 300 resistance quantitative trait loci (QTL) reported in the literature (Chen 2020; Chen and Kang 2017; Jamil et al. 2020; Tong et al. 2024; Wang et al. 2019). Using genome-wide association studies (GWAS), researchers have successfully identified genomic regions associated with YR resistance in synthetic derived wheat (Mahmood et al. 2022), CIMMYT and ICARDA breeding lines (Wu et al. 2021), landraces from China (Ye et al. 2019), pre-breeding lines derived from diverse exotic crosses (Ledesma-Ramírez et al. 2019), spring wheat (Kumar et al. 2020; Muleta et al. 2017), and winter wheat germplasm from Europe (Shahinnia et al. 2022). In recent years, variations of GWAS have emerged which provide improved efficiency and power for detecting marker-trait associations (MTAs) in crop species (Alseekh et al. 2021). A haplotype-based approach exploits the non-random associations of alleles which are inherited together and experience low levels of dissociation and rates of mutation as a result (Sunil et al. 2022). The differing combinations of tightly linked alleles present within these blocks define an individual's haplotype at that chromosomal location (Bhat et al. 2021). Some consider these blocks superior to single nucleotide polymorphisms (SNPs) when used in mapping approaches as limitations such as biallelic constraints, linkage drag, the presence of rare alleles and poor coverage can be overcome through haplotype analyses to boost genetic diversity, resulting in greater detection power and high mapping resolution across the genome (Bhat et al. 2021; Qian et al. 2017; Sunil et al. 2022). To our knowledge, haplotype-based mapping studies are lacking for YR resistance in wheat, with only three previously reported and all in relatively small pre-breeding populations (Ledesma-Ramírez et al. 2019; Mahmood et al. 2022; Wu et al. 2021). A key advantage of haplotype mapping lies in its ability to dissect the genetics of breeding populations that have undergone multiple cycles of selection. This presents significant opportunities for applying this approach to large wheat breeding populations to more accurately decipher the underlying genetic resistance.

To support effective resistance breeding into the future, it is important to identify haplotypes that are desirable in specific target environments, and those that can be used to achieve stable resistance across environments. This study aims to (1) explore phenotypic variation for YR in Australian elite wheat breeding populations across environments, (2) model genotype-by-environment (G x E) interactions using a multi-environment trial (MET) analysis, (3) identify key haploblocks associated with disease response using the local GEBV approach and (4) explore the impact of stacking resistance haplotypes *in silico* on disease response for improved YR. The outcomes of this study will support breeders in the development of cultivars with durable YR resistance across and within the target population of environments (TPEs).

## Materials and methods

### YR disease nurseries

A panel consisting of 35,986 inbred lines from the InterGrain Pty Ltd wheat breeding programme was assessed in this study. The panel is made up of commercial cultivars (Supplementary Table 1), as well as elite and legacy breeding lines that are representative of the major Australian wheat growing regions. Field experiments were conducted in artificially inoculated disease nurseries using isolates derived from endemic pathotypes present within the location of the nurseries (Supplementary Table 2). Urediniospore suspension of *P. striiformis* was prepared and loaded into calibrated ariel spray systems and sprayed with a uniform coverage pattern at tillering (Zadoks 21–30) stage during favourable environmental conditions. The field experiments were conducted at two locations: The University of Sydney Plant Breeding Institute, Cobbitty NSW (COB), and the Department of Primary Industries Farm, Horsham NSW (HRD/HOR). A total of 148 separate experiments were conducted during 2018–2022. The number of YR observations totalled to 61,262 across all environments (Supplementary Table 3) with 48 lines concurrent across the 10 environments. Experimental design was limited in the nurseries, with replication constrained to control lines, typical of commercial breeding nurseries.

All lines were assessed for their level of resistance to YR infection, which was inferred by their disease response. Disease scores were appointed for each line using a 1–9 scale (Bariana et al. 2007b), where 1 is highly resistant and 9 is highly susceptible. Scoring occurred at the peak of infection, typically in late October of each year.

### Multi-environment trial analysis

All data analyses were performed using R Core Team 2022 (R V4.2.2) (R Development Core Team 2022). A multi-environment trial (MET) analysis using a linear mixed model framework was performed on all 61,262 observations across 148 experiments and 10 environments to investigate the G x E interactions across environments using the ‘ASReml-R’ package (Butler et al. 2009). The data were analysed using the following mixed model:

$$\mathcal{y}=\mathbf{\rm X}{\varvec{\tau}}+\boldsymbol{ }\mathbf{\rm Z}\mathcal{u}+\mathcal{e}$$

$$=\boldsymbol{ }\mathbf{\rm X}{\varvec{\tau}}+\boldsymbol{ }{\mathbf{\rm Z}}_{0}{{\varvec{\upmu}}}_{0}+\boldsymbol{ }{\mathbf{\rm Z}}_{\mathcal{g}}{{\varvec{\upmu}}}_{\mathcal{g}}+\mathcal{e}$$

where $\mathbf{\rm X}$ and $\mathbf{\rm Z}$ are the design matrices associated with the fixed ${\varvec{\tau}}$ and the random $\mathcal{u}$ effects. The fixed effects include environmental main effects. Due to limited experimental designs, row and column effects were not fit as random or within a residual variance structure, as to avoid the risk of overfitting and or confounding genotypic variance that was fit as a random interaction term within environment in the model. A baseline model was generated by fitting a diagonal variance structure to the G x E interaction to conduct outlier detection and provide a comparative measure of best model fit statistics with other models. Using a studentised residual test to determine the error between values, 87 potentially problematic observations with a studentised residual of greater than 4 and less than − 4 were removed. Broad sense heritability’s (H^2^) of each experiment were calculated and those with a H^2^ of less than 0.5 were removed from the dataset. Four experiments were dropped which contained 1,343 observations in total.

The remaining 59,919 observations were subsequently run through a series of models that differ in the variance structures fit to the G x E interaction to investigate the optimal model. A diagonal base model (diag), correlation model with homogenous variation (corv), heterogeneous correlation model (corh) and factor analytic model (FA) were fitted, and resulting best model fit statistics, Akaike information criteria (AIC), Bayesian information criterion (BIC) and log-likelihood (LogLik), were compared to identify the best modelling of the G x E interactions. An FA model in the order of four hypothetical factors $\mathcal{f}$ was deemed the best model fit, where ${{\varvec{\upmu}}}_{\mathcal{g}}$ follows:

$${{\varvec{\upmu}}}_{\mathcal{g}}=\boldsymbol{ }\left({{\varvec{\lambda}}}_{1}\boldsymbol{ }\times \boldsymbol{ }{{\ell}}_{\mathcal{m}}\right){\mathcal{f}}_{1}+\dots +\boldsymbol{ }\left({{\varvec{\lambda}}}_{4}\boldsymbol{ }\times \boldsymbol{ }{{\ell}}_{\mathcal{m}}\right){\mathcal{f}}_{4}+\boldsymbol{ }{\varvec{\delta}}$$

where ${\varvec{\lambda}}$ are the known environmental loadings, $\mathcal{f}$ the hypothetical factors and ${\varvec{\delta}}$ the residuals for the model. ${{\ell}}_{\mathcal{m}}$ is the genotype variance component of the ${{\varvec{\upmu}}}_{\mathcal{g}}$ variance matrix.

### Exploring interaction classes (iClasses) for YR resistance

To explore the G x E interactions and the variation in genetic drivers across and within different environmental conditions, environmental clusters were created using interaction class (iClass) groups that refined the original 10 environments down to four environmental clusters (e.g. iClasses) with minimal cross-over G x E interactions within a cluster (Smith et al. 2021). Given that the factor analytic model proceeds in a way where the first $\mathcal{f}$ factor captures the maximum amount of genetic variance, with the second capturing the next greatest amount, and so on, iClasses can be formed based on the estimated loadings generated at each factor. Four clusters were determined using this method and are used in downstream analysis (Table 1). The formation of iClasses was restricted to three orders ($\mathcal{f}$ = 3) as minimal genetic variance was explained by the final $\mathcal{f}$ factor.

**Table 1:** The iClass environmental groupings for YR response

Interaction class (iClass)	Environments
pnn	2018_HRD, 2019_HRD, 2020_COB
pnp	2020_HRD, 2021_HRD
ppn	2019_COB, 2021_COB, 2022_HOR
ppp	2018_COB, 2022_COB

Using residual maximum likelihood (REML), the variance components of the optimal MET model were used to calculate: (1) best linear unbiased estimates (BLUEs) within each iClass leading to a BLUE per genotype per iClass, as well as (2) a corresponding Non-G x E BLUEs calculated across all 10 environments. BLUEs for all commercial cultivars tested are reported in Supplementary Table 1.

### Marker data curation and analysis of population structure

Of the unique 35,986 individuals, 20,829 of them were genotyped with 15,301 SNP markers from the Infinium™ Wheat Barley 40 K v1.0 BeadChip (Keeble-Gagnere et al. 2021). Missing calls were imputed using Beagle 5.4 (Browning et al. 2018). Data curation was conducted to remove potentially problematic markers. A total of 9,695 markers with minor allele frequencies of ≤ 1% and ≥ 10% heterozygous calls were removed. All lines had ≤ 15% heterozygosity and were within expectations for breeding material. After curation, 7,645 markers remained for downstream analysis. This process was repeated for each iClass group (ppp: 9,615 markers; ppn: 5,424 markers; pnp: 8,611 markers; pnn: 8,895 markers).

Population structure analyses were conducted using the ‘SelectionTools’ package (Selection Tools Developers 2022). Using the curated marker data, the genetic distances between lines were calculated as a genetic dissimilarity matrix using Rogers distances (Rogers 1972). The first 10 principal components (PCs) were calculated using singular value decomposition, and the variance explained by each PC was calculated using eigenvalues. A dendrogram was established using hierarchical Ward’s D clustering of the genetic distance matrix, and based on this, K-means clustering was used to define the population into four clusters.

### Haplotype mapping using the local GEBV approach

The local GEBV approach was implemented to identify key haploblocks associated with disease resistance (i.e. “resistance haplotypes”; Voss-Fels et al. 2019). A ridge-regression BLUP (rrBLUP) model was used to simultaneously predict marker effects for YR resistance across the genome to avoid potential overestimation of marker effects. Using the ‘LDHeatmap’ package (Shin et al. 2006) to visualise linkage disequilibrium (LD) across the chromosomes, a LD threshold of *r*^*2*^ 0.7 with a tolerance of three was used to define haploblocks across the chromosomes (Table 2). This threshold was selected due to the population being a breeding population with inherently high levels of relatedness, leading to strong LD signatures. It is also in line with the threshold used by Brunner et al. (2024) for haplotype-based mapping in a commercial barley breeding population. Due to computational limitations of available software packages, the Non-G x E group was found to be too large for further analysis. To move forward with analysis, highly related lines with a genetic distance of < 0.04 were removed from the Non-G x E group, resulting in a new population size of 15,291. By removing only highly related lines, this ensured that the genetic variation within the original population stayed consistent and subsequent downstream analysis was representative of the original population. Using the local GEBV method, an overall haplotype effect for each haplotype within a block was generated by taking the sum of the estimated allelic effects of each marker. The variance of effects between haplotypes within a haploblock was calculated. This variance was then min-to-max scaled, and a scaled variance threshold was used to delineate haploblocks with the highest variance.

**Table 2:** Number of haploblocks created using the local GEBV method for each iClass group

Group	Number of haploblocks
Non-G x E	4,824
ppp	4,430
ppn	3,282
pnp	4,455
pnn	4,822

### Investigating haplotype stacking in silico

To investigate the potential for wheat breeders to improve resistance levels by stacking multiple desirable haplotypes, an *in silico* analysis was performed. Haploblocks with the highest variance were selected using a scaled variance threshold of 0.25 for each iClass group, which represented the top 0.02–0.27% haploblocks in each group. Haplotypes with positive effects were not included for analysis due to the undesired effect they have on disease resistance. Lines containing all negative haplotypes (i.e. “resistance haplotypes”) with high variance were pulled for assessment, and analysis was undertaken to show the effect of “stacking” multiple resistance haplotypes on disease score. This process was repeated for each group.

## Results

### High variation for YR disease response in the elite breeding population

**Fig. 1:**
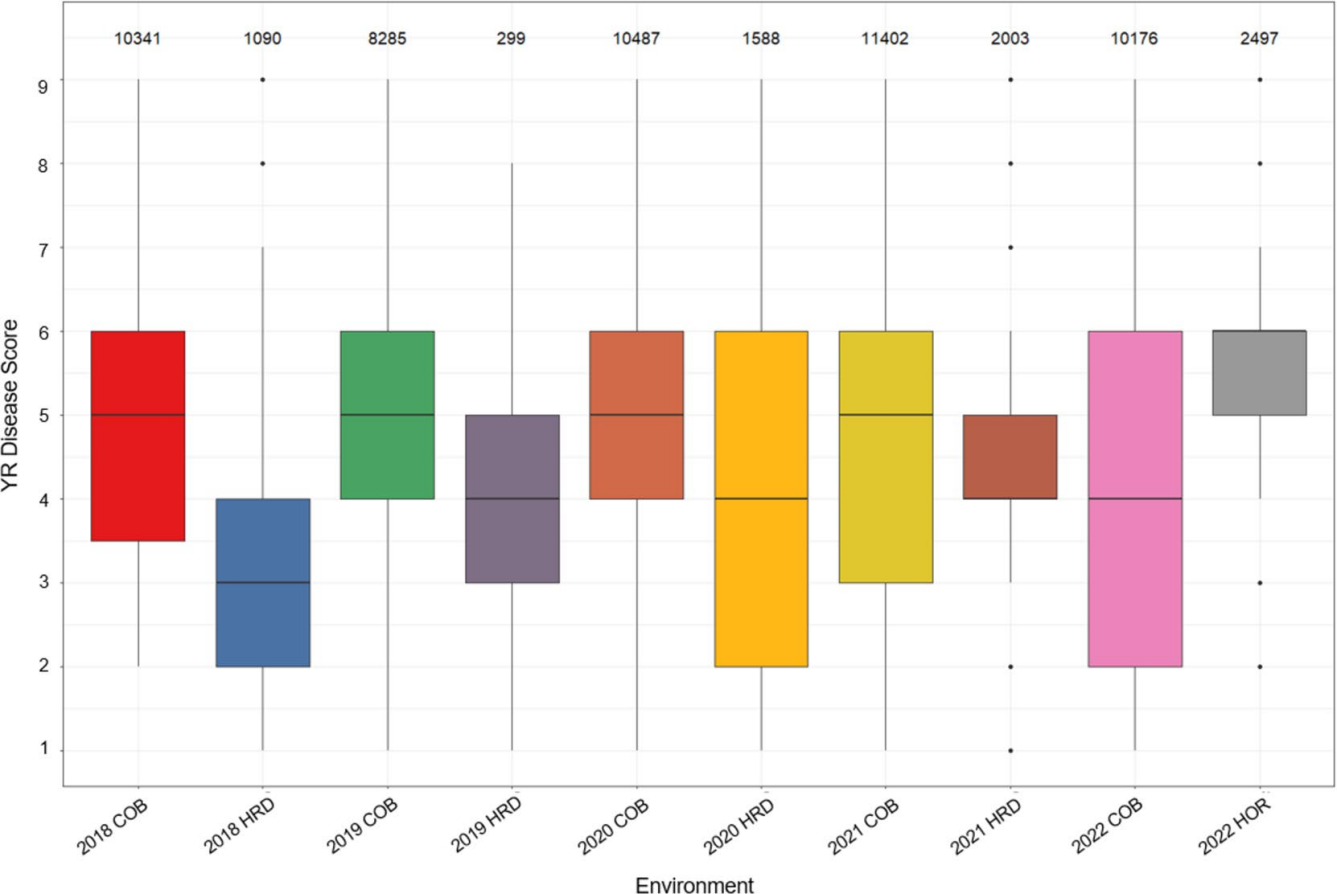
Variation in raw YR disease response across the breeding population. Box plot showing distribution of raw YR disease response on a 1–9 scale across all 10 environments with the environment code on the *x*-axis. The number of observations in each environment is displayed above each box plot

There was wide variation in disease scores across and within all 10 environments, with scores ranging from 1 to 9 (Fig. 1). A mean raw disease score between 3 and 6 was observed for all environments.

### Principal component analysis reveals moderate population structure

**Fig. 2:**
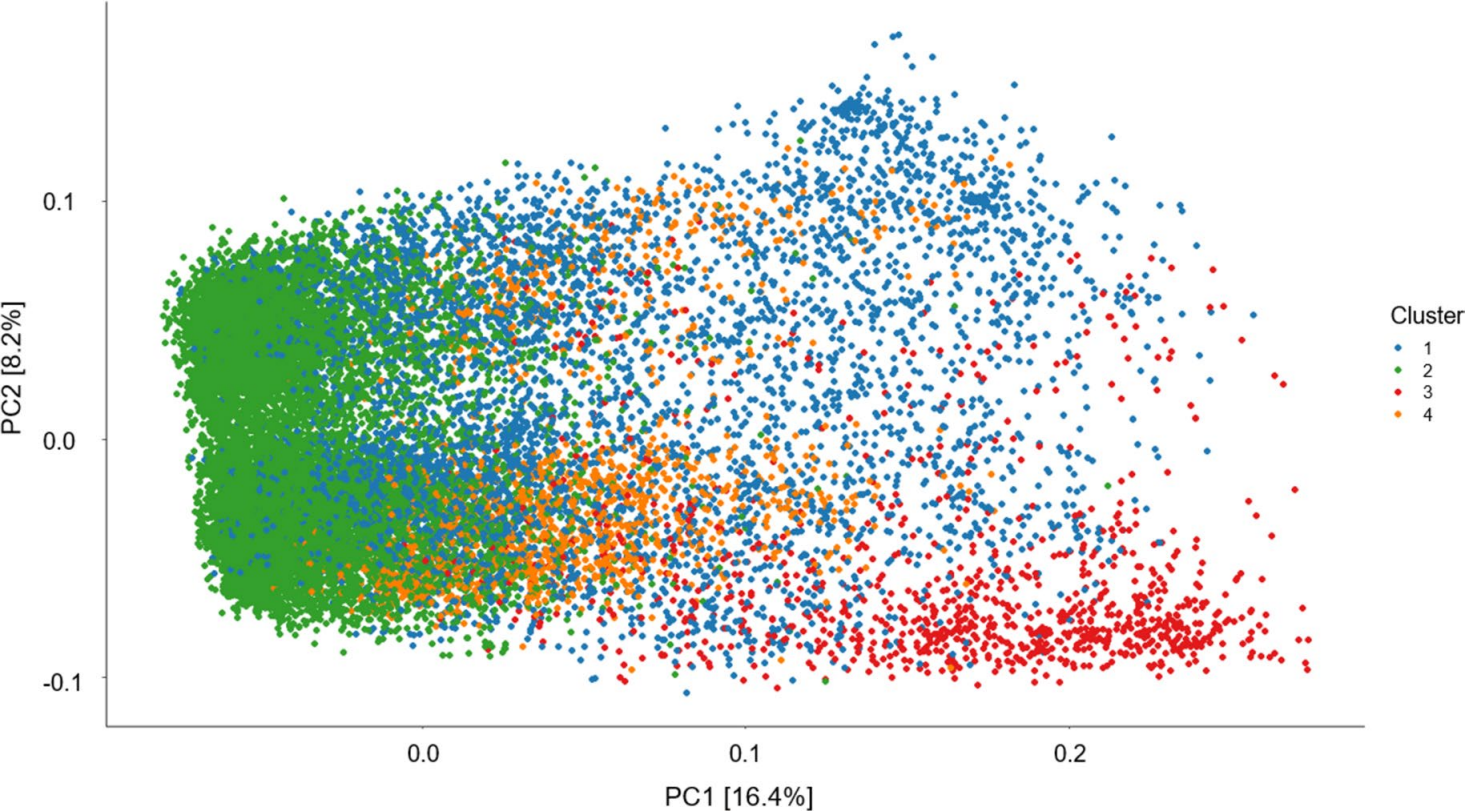
Population structure of the breeding population. Principal component analysis displaying the first and second principal components based on the genetic dissimilarity matrix and hierarchical Ward’s D clustering was used to visualise population structure and identify four main clusters within the population

A principal component analysis (PCA) on the breeding population revealed moderate structure where the first principal component (PC1) accounted for 16.4% and second principal component (PC2) accounted for 8.2%, with four distinct clusters being visualised (Fig. 2). There were general trends in clustering of lines as follows: cluster 1 consisted of a mixture of commercial cultivars adapted for both the east and west coast of Australia as well as many advanced breeding lines; cluster 2 contained predominantly commercial cultivars and breeding lines originating from the Wyalkatchem wheat variety including Mace, Scepter and Calibre; cluster 3 consisted of lines bred for the Australian Prime Hard quality market and adapted to eastern growing regions such as Gregory, Reliant and Sunprime; and lastly, cluster 4 was made up of predominately noodle wheat varieties such as Supreme, Zen and Kinsei.

### MET analysis reveals considerable genotype-by-environment interactions for YR

**Fig. 3:**
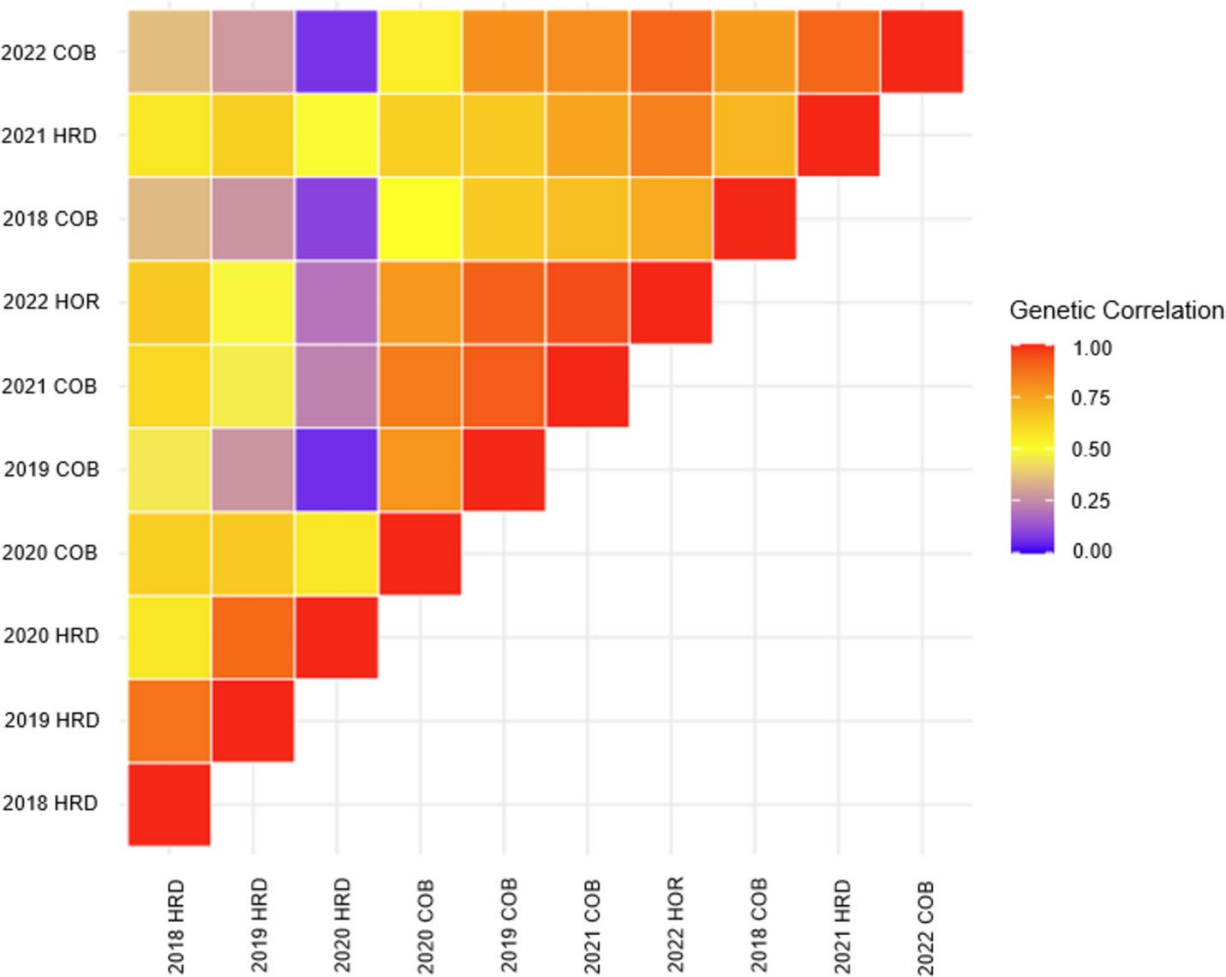
Genetic correlation matrix for YR response. Genetic correlation between environments for all 10 environments using the factor analytic 4 (FA4) model

Fitting of the baseline model showed significant crossover and scale-type G x E interactions in genotypes rankings across environments (Supplementary Fig. 1). Successive fitting of increasingly more complex variance structures from diagonal through to factor analytic 4 (FA4) saw the latter having the best AIC, BIC and Log-likelihood values and capturing a total genetic variance of 94.44% (Table 3; Supplementary Fig. 2). The FA4 model accounted for greater than 90% of the variance in 9 out of 10 environments with 2018_COB being the exception at only 60% (Supplementary Table 4). Genetic correlations between environments reinforce the presence of G x E interactions (Fig. 3). Two environments (2019_HRD and 2020_HRD) showed low correlation with other environments in the dataset with 2020_HRD showing almost no correlation with 2018_COB, 2019_COB and 2022_COB. This reinforces the presence of G x E interactions across environments within the dataset.

**Table 3:** Goodness of fit for variance structures fitted in the multi-environment trial (MET) analysis using Akaike information criteria (AIC), Bayesian information criterion (BIC) and log-likelihood (LogLik) model fit statistics

Model	AIC	BIC	LogLik	% Genetic variance captured
diag	108,728	108,827	54,353.1	NA
corv	11,0100	11,0127	55,047.1	NA
corh	105,579	105,687	52,777.6	NA
FA1	104,995	105,174	52,477.8	57.44
FA2	104,694	104,927	52,321.2	82.83
FA3	104,618	104,922	-52,275	88.38
FA4	104,616	104,616	-52,270	94.44

### Local GEBV analysis identifies 32 haploblocks associated with YR resistance

**Fig. 4:**
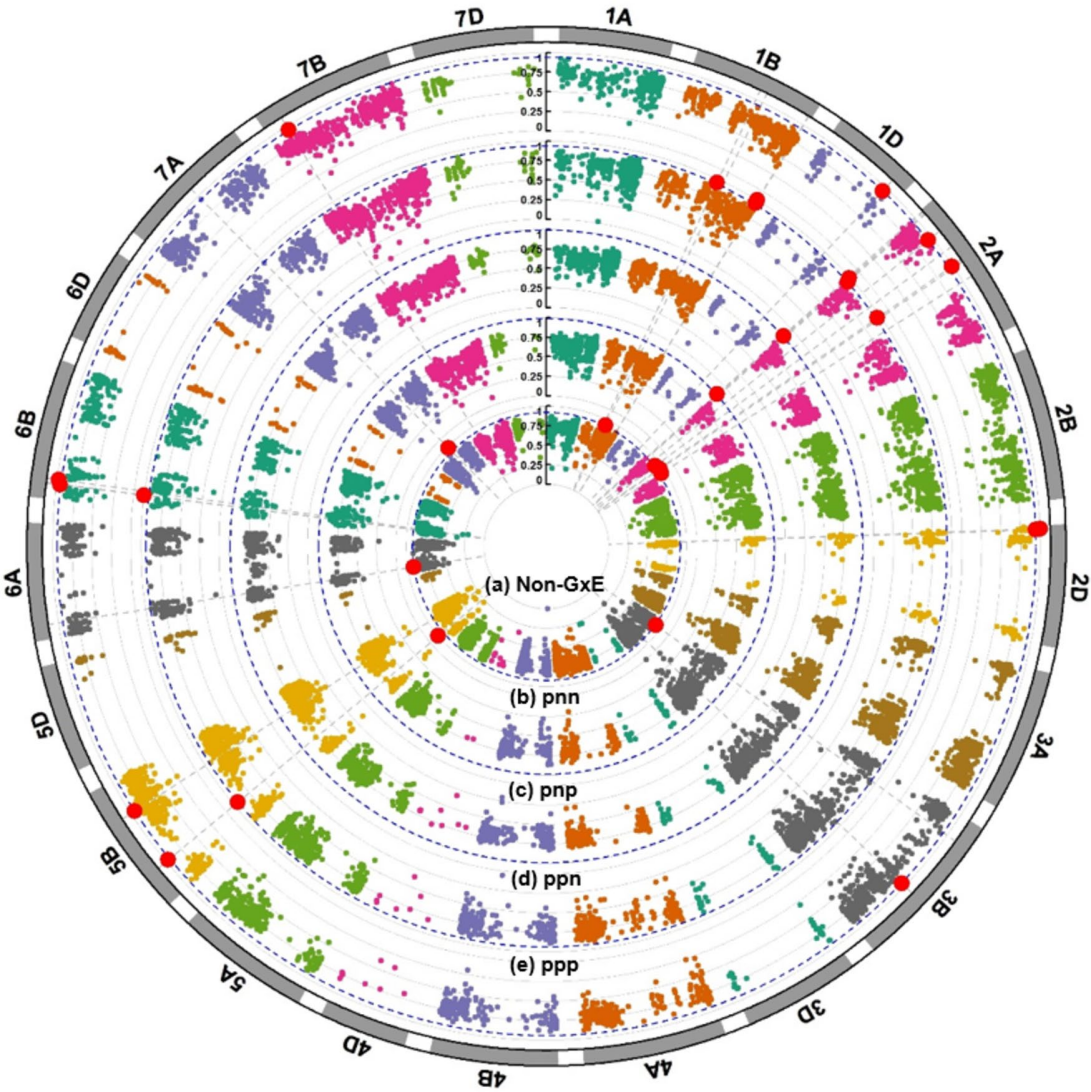
Circular Manhattan plot showing scaled variance of haploblocks for **a** Non-G x E, **b** pnn, **c** pnp, **d** ppn and **e** ppp iClass environmental clusters. The dotted blue lines represent the threshold for high-variance haploblocks that were defined by a scaled variance threshold of 0.25. The red dots indicate haploblocks that exceed the threshold and from here on are considered high-variance haploblocks (colour figure online)

A total of 32 high-variance haploblocks associated with YR response were identified across the Non-G x E and iClass groups (Fig. 4; Table 4). Twenty-three haploblocks were unique across all analysis groups, while nine were present in at least two different groups. One haploblock located on the short arm of chromosome 2A showed high variance in both pnn and ppp iClasses. Another haploblock located on the short arm of chromosome 5B showed high variance in the Non-G x E group as well as the pnn and pnp iClasses. One haploblock located on the long arm of chromosome 2A and another on the short arm of chromosome 6B both showed high variance in both pnn and pnp iClasses.

**Table 4:** High-variance haploblocks, chromosomal (Chr) positions, number of SNPs within each block, and known *Yr* gene(s) and QTLs found within each block (Tong et al. 2024)

Haploblock	Group	Chr	Variance	Start position (Mb)	End position (Mb)	Number of SNPs in haploblock	*Yr* gene(s)/QTL within haploblock
b000428	pnn	1B	0.00073	370.528469	398.093266	37	None
b000408	Non-G x E	1B	0.01113	414.606145	432.346406	26	*QYr.sicau-1B.1*^a^
b000656	pnn	1B	0.00177	677.297551	678.472909	10	*QYr.sicau-1BL*^b^
b000659	pnn	1B	0.00072	679.347052	679.347307	1	None, but *Yr29* in close proximity
b000665	pnn	1B	0.00101	682.638989	683.057292	2	None, but *Yr29* in close proximity
b000669	pnp	1D	0.00087	410.184427	418.752625	17	None
b000531	ppn	2A	0.03358	5.814657	25.671321	1	*QYr.sicau-2AS*^b^; *QYr.hebau-2AS*^c^; *QYrtb.orz-2AS*^d^; *QYr.lrdc-2A.1*^e^; *Qyr.gaas.2A*^f^
b000747	pnn	2A	0.00141	**3.158396**	23.807392	13	*QYr.sicau-2AS*^b^; *QYr.hebau-2AS*^c^; *QYrtb.orz-2AS*^d^; *QYr.lrdc-2A.1*^e^
b000753	ppp	2A	0.05495	**3.158396**	23.807392	13	*QYr.sicau-2AS*^b^; *QYr.hebau-2AS*^c^; *QYrtb.orz-2AS*^d^; *QYr.lrdc-2A.1*^e^
b000750	pnn	2A	0.00076	24.837595	25.671321	1	*Qyr.gaas.2A*^f^
b000753	pnn	2A	0.00062	28.495187	28.714144	1	None, but *Yr17* in close proximity
b000771	pnp	2A	0.00091	128.813111	165.836721	26	None
b000781	Non-G x E	2A	0.01435	211.363047	297.512806	34	*QYr.cim-2AS*^g^; *QYrpd.swust-2AS*^h^; *QYr.uga-2AS*^i^
b000840	pnn	2A	0.00082	**211.986415**	474.906026	114	*QYr.cim-2AS*^g^; *QYrpd.swust-2AS*^h^; *QYr.uga-2AS*^i^; *QYrsn.nwafu-2AS.2*^j^; *QYr.inra-2AL*^k^; *QYrns.orz-2AL*^d^
b000780	pnp	2A	0.00213	**211.986415**	474.906026	115	*QYr.cim-2AS*^g^; *QYrpd.swust-2AS*^h^; *QYr.uga-2AS*^i^; *QYrsn.nwafu-2AS.2*^j^; *QYr.inra-2AL*^k^; *QYrns.orz-2AL*^d^
b000782	Non-G x E	2A	0.02747	298.247448	412.228769	53	*QYrsn.nwafu-2AS.2*^j^; *QYr.inra-2AL*^k^
b000783	Non-G x E	2A	0.01818	415.017642	474.906026	27	*QYrns.orz-2AL*^d^
b001308	pnp	2D	0.00306	15.938754	16.251731	1	None
b001309	pnp	2D	0.00255	16.251731	17.386862	2	*Yrq1*^l^
b001310	pnp	2D	0.00084	20.40272	20.404906	6	None
b001887	Non-G x E	3B	0.01218	129.307759	137.543341	16	*QYr.nafu-3BS*^m^; *QYr.uga-3BS.2*^i^
b001827	pnp	3B	0.00097	487.843768	509.959695	34	*QYrPI197734.wgp-3B*^n^
b003172	Non-G x E	5B	0.07297	**120.310899**	243.494347	42	*QYrPI181410.wgp-5BL.2*°; *QYr.cim-5BL*^p^
b003171	pnn	5B	0.00054	**120.310899**	243.494347	42	*QYrPI181410.wgp-5BL.2*°; *QYr.cim-5BL*^p^
b002949	pnp	5B	0.00144	**120.310899**	243.494347	42	*QYrPI181410.wgp-5BL.2*°; *QYr.cim-5BL*^p^
b003059	pnp	5B	0.00087	499.252885	518.343702	25	*QYr.caas-5BL.1*^q^
b003624	Non-G x E	6A	0.01018	69.729083	78.888712	16	None
b003778	pnn	6B	0.00172	**67.346657**	90.643761	20	*QYr.sicau-6BS*^b^; *QYr.caas-6BS.2*^r^
b003500	pnp	6B	0.001	**67.34671**	95.333926	20	*QYr.sicau-6BS*^b^; *QYr.caas-6BS.2*^r^
b003501	pnp	6B	0.00173	98.000683	113.948677	18	*QYrsk.wgp-6B*^s^
b004225	Non-G x E	7A	0.04602	292.294676	452.448374	53	*Yr75*^t^
b004055	pnp	7B	0.00094	108.282808	110.7225	13	None

*Bold positions represent haploblocks with shared chromosomal positions. Flanking markers for haploblocks can be obtained using the physical position and the Infinium™ Wheat Barley 40 K v1.0 BeadChip (Keeble-Gagnere et al. 2021)

^a^ Ma et al. 2019; ^b^ Wang et al. 2022; ^c^ Zhang et al. 2019; ^d^ Vazquez et al. 2015; ^e^ Farzand et al. 2022; ^f^ Cheng et al. 2022; ^g^ Calvo-Salazar et al. 2015; ^h^ Zhou et al. 2022; ^i^ Hao et al. 2011; ^j^ Huang et al. 2021; ^k^ Mallard et al. 2005; ^l^ Cao et al. 2012; ^m^ Zhou et al. 2015; ^n^ Liu et al. 2020a; ^o^ Liu et al. 2020b; ^p^ Yang et al. 2013; ^q^ Lu et al. 2009; ^r^ Ren et al. 2012; ^s^ Liu et al. 2019; ^t^ Yao et al. 2022

Only one haploblock (b004225) from the Non-G x E group was found to contain a known *Yr* gene, *Yr75* (Table 4). Twenty-two haploblocks contained known YR resistance QTLs located on chromosomes 1B, 2A, 2D, 3B, 5B and 6B (Table 4). Nine haploblocks located on chromosomes 1B, 1D, 2A, 2D, 6A and 7B did not contain any known *Yr* genes or QTL and thus were considered novel (Table 4).

**Fig. 5:**
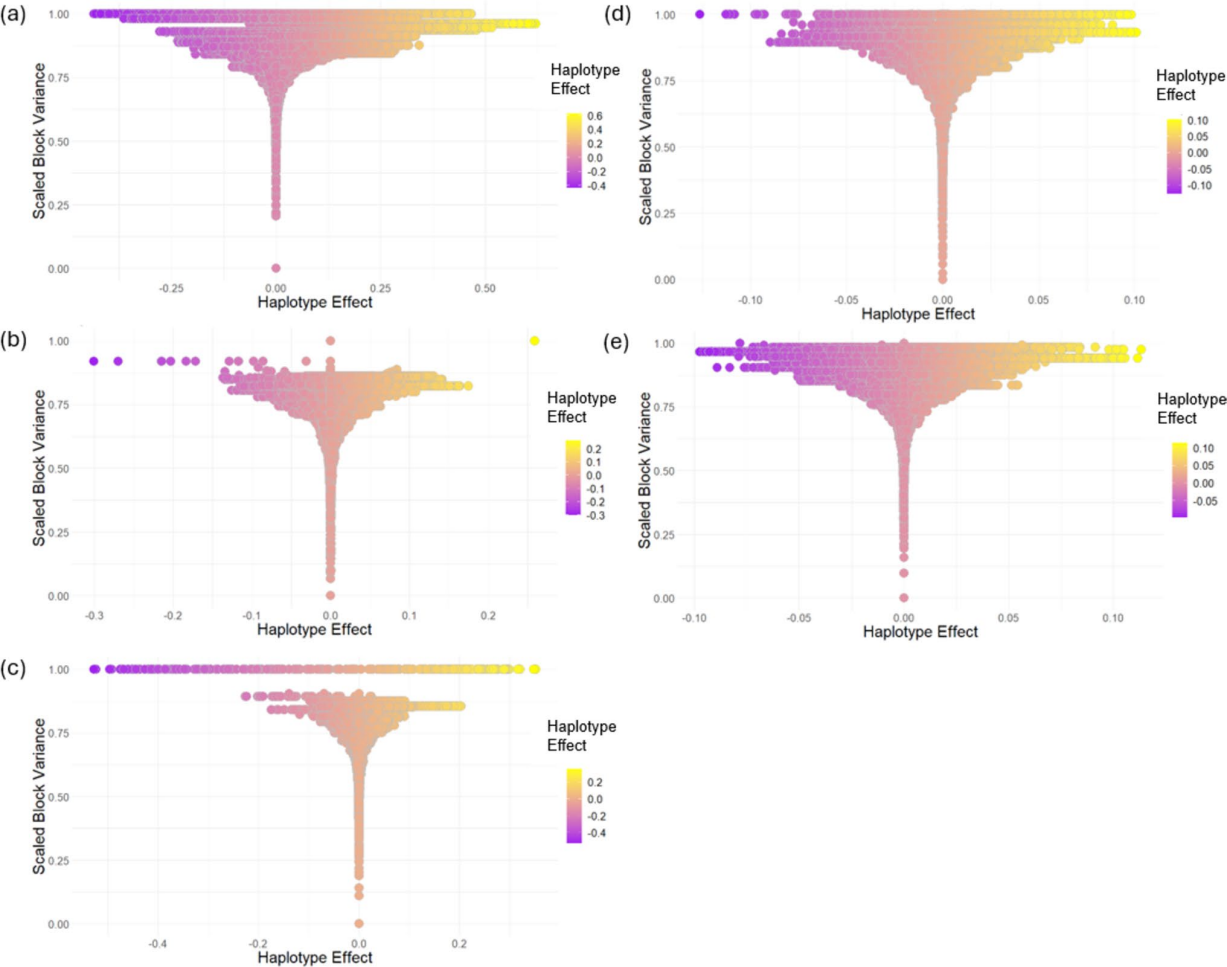
The relationship between scaled block variance and haplotype effects at each haploblock within **a** Non-G x E, **b** ppn, **c** ppp, **d** pnn and **e** pnp iClass environmental clusters. Each dot represents a unique haplotype within the haploblock in the wheat breeding population

To explore the effects of each haplotype within a block across the groups, scaled block variance graphs were generated (Fig. 5). The majority of groupings show an even distribution of positive and negative effect haplotypes, except ppn and ppp, which are skewed towards negative effects. Haploblocks selected for downstream analysis were those with the highest scaled variances.

### Stacking of resistance haplotypes can improve disease resistance for YR

Resistance haplotypes were “stacked” *in silico* to investigate their cumulative effect on YR disease response. Haplotype stacking was explored for groups of environments that showed multiple high-variance haploblocks contributed to resistance. As the ppp and ppn iClasses demonstrated only one haploblock with high variance, which had haploblock effects ranging from approximately − 0.5–0.3 and 0–0.2, respectively, these iClasses were not subject to haplotype analysis as stacking of multiple resistance haplotypes *in silico* could not be explored (Fig. 5b-c).

**Fig. 6:**
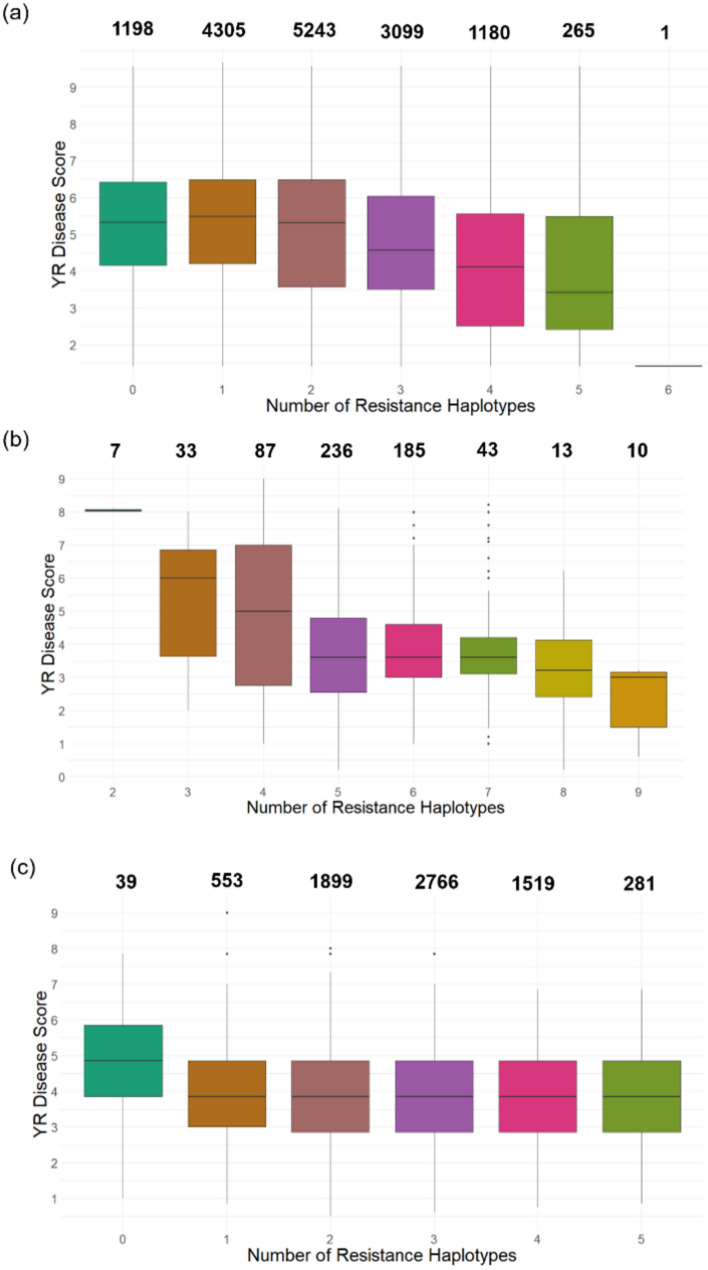
Stacking of resistance haplotypes and their effect on YR disease score for the **a** Non-G x E, **b** pnp and **c** pnn iClass groups. The YR disease score is the BLUEs estimated from the MET analysis using measured YR scores. The resistance haplotypes in **b** and **c** are predominately iClass group specific except where resistance is shared across groupings. The x-axis reflects the number of resistance haplotypes within the population for the iClass analysis group, where the absence of 0 or 1 resistance haplotypes means the scale will start at the minimum number (e.g. 2 haplotypes in (**b**)). The number of individuals that fall within each number of resistance haplotypes grouping is detailed at the top of each boxplot (colour figure online)

For the Non-G x E group, lines with zero to two resistance haplotypes had a moderately resistant/moderately susceptible average disease score of between 5 and 6. Lines with three or more resistance haplotypes showed a linear improvement in average disease resistance as more resistance haplotypes accumulated in the wheat lines. A single line with six resistance haplotypes showed a very resistant phenotype with a disease score of 1 (Fig. 6a). Similarly, for the pnp iClass, the disease score reduced in a linear pattern as more resistance haplotypes accumulated, with those lines containing nine haploblocks having an average disease score of 3 (Fig. 6b). Within the pnn iClass, no linear relationship was observed as resistance haplotypes accumulated within a line, with six haploblocks showing the same effect on average disease score as those with only one (Fig. 6c).

### Sources of resistance haplotypes in Australian elite wheat breeding germplasm

**Fig. 7:**
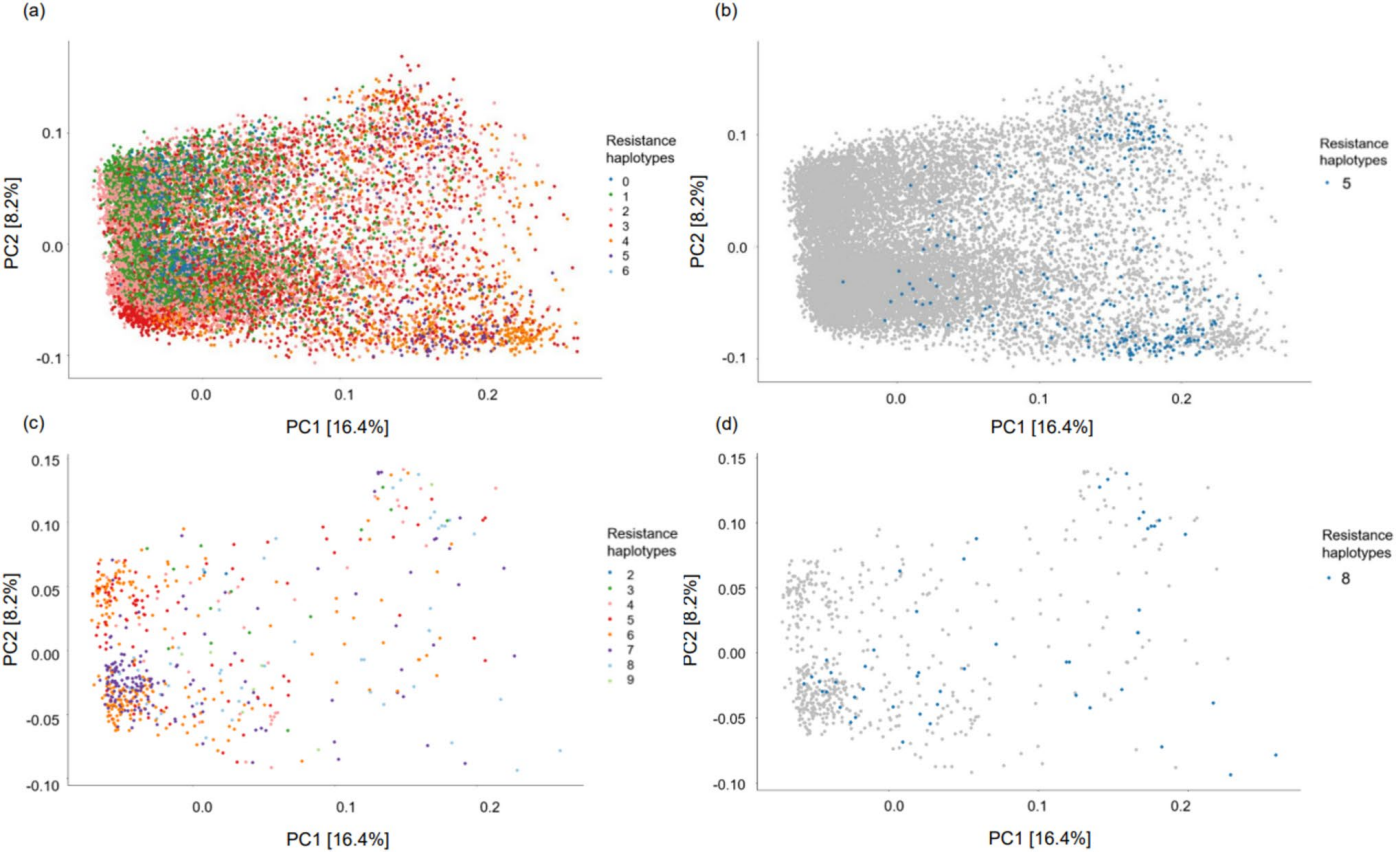
Resistance haplotype distribution for the Non-G x E and pnp groups. **a** PCA of the dissimilarity matrix of the Non-G x E population displaying the first and second PCs where genotypes are coloured according to the number of key resistance haplotypes they carry and **b** the distribution of genotypes that carry five resistance haplotypes highlighted in blue. **c** PCA of the pnp iClass displaying the first and second PCs where genotypes are coloured according to the number of key resistance haplotypes they carry and **d** the distribution of genotypes that carry eight resistance haplotypes highlighted in blue (colour figure online)

To provide insight into the origin and distribution of resistance haplotypes, population structure of the Non-G x E group and the lines carrying these haplotypes were explored (Fig. 7a). There was only one line that carried six resistance haplotypes which was determined to be an advanced breeding line. Those lines that carried five resistance haplotypes tended to fall within cluster 1 and cluster 3 which included predominately commercial cultivars and Australian Prime hard quality breeding lines, respectively (Fig. 7b). The pnp iClass was also analysed due to the strong negative trend observed during haplotype stacking (Fig. 6b). Notably, all lines with six or more resistance haplotypes were advanced breeding lines, with the exception of a cultivar originating from China (Fig. 7c). The lines that carried eight resistance haplotypes were spread across the clusters (Fig. 7d).

## Discussion

### The importance of genotype-by-environment-by-pathotype (G x E x P) interactions for durable and stable resistance

To address the resistance breakdown observed with ASR genes, breeders have shifted their focus towards selection for novel APR genes, essential for durable YR resistance. It is well known that APR genes are strongly influenced by the environment (Hickey et al. 2012; Singh et al. 2000). To that point, we have identified a notable amount of variation in disease response and re-ranking of genotype performance, both scale and crossover G x E interactions, across the 10 environments within this study (Supplementary Fig. 1 and Supplementary Fig. 2). We hypothesise that environmental factors such as cool night temperatures and high humidity may be key drivers for the variation observed across environments, as infection and disease progression are highly dependent on favourable weather conditions. As in drought research, where environmental characterisation relies on modelling the degree of water stress experienced in different environment clusters, a similar characterisation of disease nurseries could be adopted to support the interpretation of G x E interactions, including those involving APR. Yet, much of the YR research does not acknowledge the environmental factors that drive differences in APR expression, likely a result of the complexity of environmental modelling for quantitative disease traits.

In our study, modelling G x E interactions for YR resistance was a core focus, where we employed the iClass method to group nurseries into clusters that minimise crossover G x E interactions. Here, we assume the environmental drivers and pathotypes at the nurseries within an iClass group are similar yet differ across groupings. Exploration of the year-location combination within each iClass showed that environments within the same geographic location tended to cluster together. For example, the pnn and pnp iClasses contained all the nurseries at Horsham (with the exception of 2022_HOR; Table 1) and were separated from nurseries at Cobbitty found in ppn and ppp with the exception of 2020_COB. Furthermore, haplotype mapping within the ppn and ppp iClasses only detected one high-variance haploblock on chromosome 2A. This suggests that they are more similar to each other than other iClasses, which is consistent with their positive loadings for the first two factors (i.e. “pp”). This also highlights the importance of the first two factors, particularly the second factor, that may be reflective of the differing virulence factors within the pathotypes. Notably, environments that were inoculated with a pathotype virulent for *Yr27* tend to have “pp” as their first two factors, while those with a pathotype virulent for *Yr33* have “pn” (Supplementary Table 1). Neither of these APR genes were detected in the study; however, both originate from wild sources and may not be present in the breeding population, suggesting other GxP interactions could be influencing the iClass groupings.

Due to the nature of commercial breeding datasets, this study was constrained by the lack of detailed weather and soil information for each of the rust nurseries. Further, the datasets constituting each iClass are unbalanced, where all individuals were not tested in each iClass. Unbalanced data are a highly common feature of real-world plant breeding data, but this is often counteracted through analyses by exploiting the high degree of genetic relatedness within the breeding population. As a result, we can only speculate on the environmental factors that are driving the differences observed in disease response across iClasses. Consequently, it is highly recommended that future research involving G x E x P modelling should incorporate as much weather and soil data, along with detailed pathotype information, as possible. Advanced mixed modelling can then be used to provide greater insight into environmental variables and pathotype features driving YR resistance, where knowledge gained could be incorporated into the design of wheat varieties with custom resistance packages for target environments. These bespoke resistance packages are integral in providing robust resistance amidst rising temperatures and increasing seasonal variability.

### A path to enduring resistance through targeting stability and specificity

Notably, only one high-variance haploblock, b004225, was found to contain *Yr75*, an APR gene located on the long arm of chromosome 7A (Table 4), which was originally mapped using a recombinant inbred population derived from two cultivars, Axe and Nyabing-4 (Kanwal et al. 2021). The lack of alignment with known resistance genes is somewhat not surprising given the majority of gene discovery research has focussed on mapping new sources of resistance in landraces and wild relatives, rather than elucidating the genetic basis of resistance within elite breeding populations. Two resistance genes common in Australian wheat breeding, *Yr29* and *Yr17* (Park et al. 2022), were found to be in close proximity to b000659 on chromosome 1B and b000753 (within pnn iClass) on 2A, respectively. All pathotypes used were virulent to *Yr17*; therefore, it is unlikely that b000753 is *Yr17*. Previously reported rust resistance QTL were also identified in 22 of the high-variance haploblocks (Table 4), and of these there were a mixture of both ASR and APR sources, while APR QTL predominated. This result is unsurprising and consistent given the shift in breeding and selection for APR. Seven out of the 32 high-variance haploblocks did not contain or were not in close proximity to, previously reported sources of *YR* resistance, highlighting their potential as novel resistance sources. Interestingly, all but one of the novel haploblocks were deemed environment specific, further reinforcing the power of the mixed model approach to account for and tease apart G x E interactions and the environmental sensitivity of the genetic drivers.

In contrast, some haploblocks were located at the same chromosomal position across iClasses and could represent environmentally stable resistance. As an illustration, four separate high-variance haploblocks were common across one or more iClasses, one haploblock on the short arm and one on the long arm of chromosome 2A, one on chromosome 5B and one chromosome 6B (Table 4). This suggests that the resistance conferred by haplotypes at these haploblocks has high environmental stability and is obvious breeding targets for stable resistance. The strategic choice to prioritise stable or environment-specific resistance sources depends heavily on the breeding approach. However, the identification of both types of haplotypes offers breeders the flexibility to tailor resistance profiles, ultimately fostering the deployment of resistant varieties across diverse environments.

### Complexity of APR

Many agriculturally important traits, such as disease resistance, are quantitative in nature and are controlled by many small effect genes across the genome. Here, our *in silico* analyses have shown that stacking resistance haplotypes can rapidly increase levels of YR resistance. One such example comes from the Non-G x E and pnp iClasses, where a negative linear trend was observed between disease score and the accumulation of resistance haplotypes within a line, such that as resistance haplotypes increased the disease score decreased (Fig. 6a–b). Previous studies have shown that some resistance genes can interact with one another through additive or epistasis effects to enhance overall effectiveness (Wang et al. 2023; Zheng et al. 2017), thus utilising a haplotype-based mapping approach to identify resistance may better capture these effects *in vitro*, providing better insight into optimal resistance gene combinations. Notably, genotypes with five or more of the high-variance resistance haplotypes were either commercially released cultivars or advanced breeding lines targeting the Australian Prime Hard wheat quality market, typically grown in the northern growing regions of Australia (Fig. 7). This suggests that some of the most influential sources of YR resistance are already present and available within elite breeding germplasm, enabling rapid and relatively undisruptive stacking. However, it is important to note that the linear trend for stacking resistance haplotypes was not observed in all iClasses, and despite the favourable effects of resistance haplotypes on the disease phenotype, we did not see a uniform trend. To illustrate this, a slight decrease in average disease scores was observed in the pnn iClass, when a line carried one resistance haplotype yet showed no further improvement as the number of resistance haplotypes increased (Fig. 6c). Therefore, it is hypothesised that, despite these resistance haplotypes having high haploblock variances, these loci do not act alone to confer resistance, and it is likely that a combination of low and high-variance haploblocks is important. Furthermore, the lack of trend could be influenced by a number of interaction factors, such as interaction effects between different haplotype combinations leading to enhanced or diminished resistance. In addition, G x E and genotype-by-pathogen interactions that were not captured in the study’s G x E modelling along with functional differences between haploblocks may lead to variability in their contributions to resistance. Furthermore, this study focused exclusively on APR, while ASR and the combination of both types of resistance can be important in certain environments and pathotype combinations. Due to the complexity in the genetic architecture underlying YR resistance, traditional breeding approaches, such as marker-assisted selection, are likely unfeasible and it is probable that the most suitable approach to assess genetic merit is a whole-genome method, such as genomic prediction, targeting both ASR and APR. By utilising the whole-genome haplotype effects generated in this study, the “ultimate genotype” (Hayes et al. 2024; Kemper et al. 2012) can be simulated *in silico* for either stable or environment-specific resistance. This “ultimate genotype” is distinct only to the examined population, yet it enables critical benchmarking of the genetic diversity within the population and illuminates the opportunity for introgression of new sources of resistance, which can be achieved through novel parental selection approaches, such as that described in Villiers et al. (2024).

In this study, the integration of advanced mixed modelling for G x E interaction analysis and haplotype-based mapping has successfully enabled a comprehensive dissection of genomic regions associated with both stable and environment-specific YR resistance within Australian wheat breeding germplasm. Furthermore, this is the largest study to date exploring YR resistance in wheat and has identified optimal breeding tools and approaches to support the development of varieties with robust rust resistance across environments and seasons, crucial for future crop improvement.

## Supplementary Information

Below is the link to the electronic supplementary material.

Supplementary file1 (XLSX 24 KB)

Supplementary file2 (DOCX 848 KB)

## Data Availability

The data that support the findings of this study are available from InterGrain Pty Ltd, but restrictions apply to the availability of these data, which were used under licence for the current study, and so are not publicly available. Data are however available from the authors upon reasonable request and with permission of InterGrain Pty Ltd.
