# Supplementary material for: A large-scale multi-environment study dissecting adult-plant resistance haplotypes for stripe rust resistance in Australian wheat breeding populations
Source: Theor Appl Genet. 2025 Mar 13;138(4):72. doi: 10.1007/s00122-025-04859-2 (PMC11906565; doi:10.1007/s00122-025-04859-2)
Supplement: Supplementary file 2 — Supplementary file2 (DOCX 848 KB) [file 122_2025_4859_MOESM2_ESM.docx]

**Supplementary Table 1**

Commercial cultivars and BLUEs across environmental clusters. See associated excel file.

**Supplementary Table 2**

| **Environment** | **Pathotype** |
| --- | --- |
| 2018_COB | 134 E16+Yr17+Yr27+, 199 E76A-, 110 E143A+ |
| 2018_HRD | WA+17 and 239 E237 A-17+33+ |
| 2019_COB | Culture 674 |
| 2019_HRD | 198E16A+J+T+17+ / 239 E237 A- 17+ 33+ /238 E191 A+ 17+ 33+ |
| 2020_COB | 34E16A+Yr17+Yr27+ |
| 2020_HRD | 198E16A+J+T+17+ / 239 E237 A- 17+ 33+ /238 E191 A+ 17+ 33+ |
| 2021_COB | 198 E16 A+ J+ T+ 17+ (687) and traces of 239E237A- 17+33+ (674) |
| 2021_HRD | 198 E16 A+ J+ T+ 17+ and 239 E237 A- 17+ 33+ |
| 2022_COB | 134 E16 A+ Yr17+ Yr27+ [=617],198 E16 A+ J+ T+ [=687],110 E143 A+ [=444],239 E237 A- 17+ 33+ [=674] |
| 2022_HOR | 239 E237 A- 17+ 33+ |

Summary of YR pathotypes used in each environment of the study.

**Supplementary Table 3**

Data summary of wheat lines used in the study.

| **Total stripe rust observations** | 61,262 |
| --- | --- |
| **Environments** | 10 |
| **Experiments** | 148 |
| **Years** | 5 (2018-2022) |
| **Locations** | 2: Cobbity (COB) and Horsham (HRD/HOR) |
| **Unique lines** | 35,986 |
| **Total inbred lines genotyped** | 20,829 |

**Supplementary Table 4**

Percentage of genetic variance captured by each factor for each environment and the total percentage of genetic variance captured for each environment using the factor analytic model 4 (FA4) model.

| **Environment** | **Factor 1** | **Factor 2** | **Factor 3** | **Factor 4** | **% Genetic variance** |
| --- | --- | --- | --- | --- | --- |
| **2018_COB** | 54.03473 | 4.492467 | 0.953304 | 0.144336 | 59.62483 |
| **2018_HRD** | 42.68133 | 25.44565 | 14.39367 | 17.47935 | 100 |
| **2019_COB** | 74.03907 | 6.95202 | 8.238781 | 4.069538 | 93.29941 |
| **2019_HRD** | 35.01067 | 61.18684 | 0.082079 | 3.720411 | 100 |
| **2020_COB** | 67.03674 | 8.503652 | 9.478739 | 14.98087 | 100 |
| **2020_HRD** | 14.18862 | 80.21865 | 3.531983 | 2.060744 | 100 |
| **2021_COB** | 86.56146 | 0.907584 | 6.514089 | 1.191817 | 95.17495 |
| **2021_HRD** | 85.96334 | 0.992575 | 12.06212 | 0.981969 | 100 |
| **2022_COB** | 82.84522 | 11.58663 | 5.274269 | 0.293882 | 100 |
| **2022_HOR** | 94.91884 | 2.0286 | 2.59241 | 0.460151 | 100 |

**Supplementary Fig. 1**


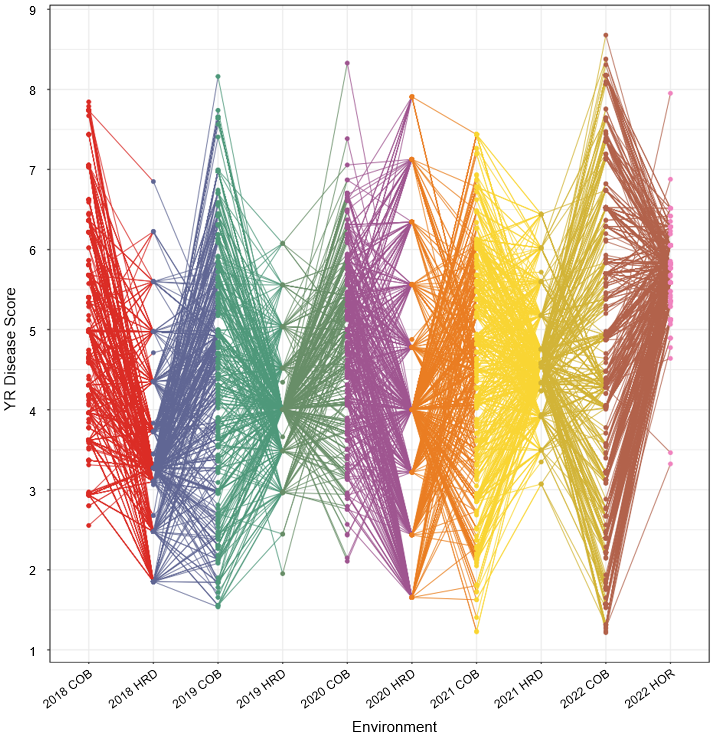


Best linear unbiased predications (BLUPs) for stripe rust (YR) disease response for all 10 environments derived from multi-environment trial (MET) analysis using the base diagonal model.

**Supplementary Fig. 2**

**
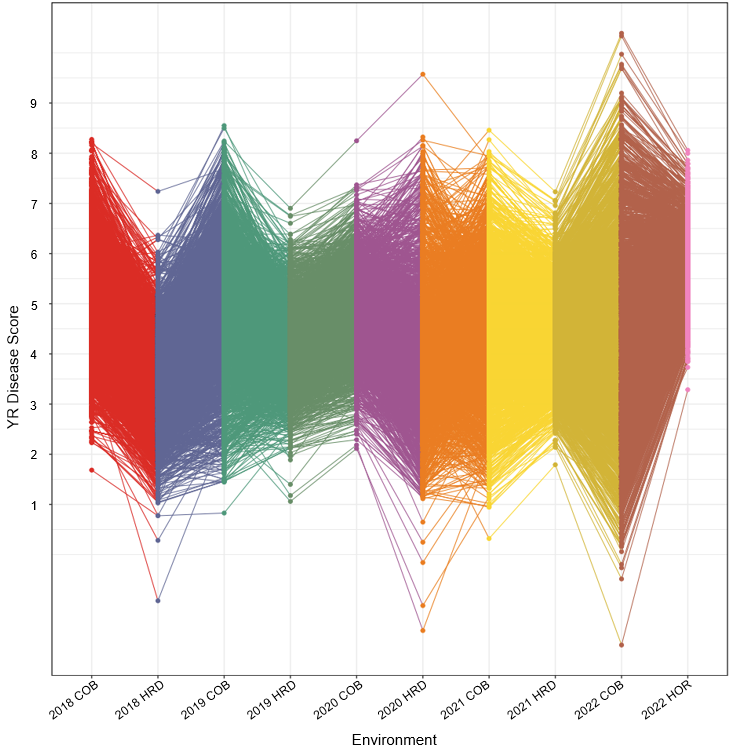
**

Best linear unbiased predications (BLUPs) for stripe rust (YR) disease response for all 10 environments derived from multi-environment trial (MET) analysis using the factor analytic (FA) model at order four where the performance is predicted for all genotypes at each environment, whether physically present or not.
